# Biomarker discovery for chronic liver diseases by multi-omics – a preclinical case study

**DOI:** 10.1038/s41598-020-58030-6

**Published:** 2020-01-28

**Authors:** Daniel Veyel, Kathrin Wenger, Andre Broermann, Tom Bretschneider, Andreas H. Luippold, Bartlomiej Krawczyk, Wolfgang Rist, Eric Simon

**Affiliations:** 1 Boehringer Ingelheim Pharma GmbH & Co. KG, Drug Discovery Sciences, Birkendorfer Str. 65, D-88397 Biberach Riss, Germany; 2 Boehringer Ingelheim Pharma GmbH & Co. KG, CardioMetabolic Diseases Research, Birkendorfer Str. 65, D-88397 Biberach Riss, Germany; 3 Boehringer Ingelheim Pharma GmbH & Co. KG, Computational Biology, Birkendorfer Str. 65, D-88397 Biberach Riss, Germany

**Keywords:** Proteomics, Transcriptomics, Biomarkers, Liver diseases

## Abstract

Nonalcoholic steatohepatitis (NASH) is a major cause of liver fibrosis with increasing prevalence worldwide. Currently there are no approved drugs available. The development of new therapies is difficult as diagnosis and staging requires biopsies. Consequently, predictive plasma biomarkers would be useful for drug development. Here we present a multi-omics approach to characterize the molecular pathophysiology and to identify new plasma biomarkers in a choline-deficient L-amino acid-defined diet rat NASH model. We analyzed liver samples by RNA-Seq and proteomics, revealing disease relevant signatures and a high correlation between mRNA and protein changes. Comparison to human data showed an overlap of inflammatory, metabolic, and developmental pathways. Using proteomics analysis of plasma we identified mainly secreted proteins that correlate with liver RNA and protein levels. We developed a multi-dimensional attribute ranking approach integrating multi-omics data with liver histology and prior knowledge uncovering known human markers, but also novel candidates. Using regression analysis, we show that the top-ranked markers were highly predictive for fibrosis in our model and hence can serve as preclinical plasma biomarkers. Our approach presented here illustrates the power of multi-omics analyses combined with plasma proteomics and is readily applicable to human biomarker discovery.

## Introduction

Nonalcoholic fatty liver disease (NAFLD) is the major liver disease in western countries and is often associated with obesity, metabolic syndrome, or type 2 diabetes. Around 10% of NAFLD patients successively develop nonalcoholic steatohepatitis (NASH)^1^, which is characterized by hepatic inflammation and fibrosis^2^. NASH is projected to be the major reason for liver transplantation globally by 2020^3^, because it can further progress to liver cirrhosis and/or liver cancer. Although there is some progress towards a better disease understanding, there is no approved drug available to treat NASH patients^4^. One major hurdle to develop novel drugs is the lack of non-invasive clinical biomarkers. Currently, liver biopsies are the gold standard for diagnosis and disease staging^2,5^. The apparent problem of liver biopsies is their invasiveness and variability resulting from the limited sample size in combination with the heterogeneity of the disease pathology over the whole organ. Therefore, non-invasive biomarkers supporting diagnosis, monitoring therapeutic efficacy and disease progression are highly desired.

NASH and liver fibrosis are accompanied by massive cellular transformations, e.g. hepatocyte ballooning and apoptosis, and extracellular matrix (ECM) deposition by hepatic stellate cells. Thus, specific molecules from hepatocyte leakage and/or the ECM might be detectable in plasma like the generic liver damage markers alanine aminotransferase and aspartate aminotransferase. Existing soluble biomarkers for NASH and fibrosis cover specific disease aspects like inflammation, fibrosis, apoptosis, and oxidative stress, but are not used routinely^5^. For example, the ELF score represents a panel of three biomarkers related to ECM deposition (PIIINP, TIMP1, and Hyaluronic acid), recommended for screening NAFLD patients for advanced fibrosis^6^. Although showing moderate to excellent predictive accuracy, the ELF test lacks sensitivity for early fibrotic stages^5^.

Since NASH is a heterogeneous disease, biomarkers for e.g. monitoring treatment response will likely depend on the specific mode of action of a drug^5,7^. To address this, several studies used metabolomics approaches to discover soluble biomarker candidates for NASH^8–11^. Other studies used plasma protein profiling to identify proteins associated with NAFLD and Cirrhosis^12,13^ or to generate a steatosis classifier together with genetic and clinical parameters^14^. However, there is a lack of studies that systematically asses the translation of the molecular changes in the diseased liver at the RNA level to changes at the liver and plasma protein level for the discovery of non-invasive biomarkers.

Similar as for the human disease, preclinical *in vivo* models rely on the terminal histopathological and molecular assessment of liver material. Consequently, it is difficult to monitor longitudinal disease progression and therefore estimate the right time-point to evaluate the efficacy of a test compound in a subchronic experiment. There are several preclinical animal models for NASH established or under development^15–17^. They differ in the way of triggering a NASH-like phenotype (obesogenic dietary, nutrient-deficient dietary, genetic, chemically induced, surgery-based) and in their ability to reflect the human etiology and histopathology^15^. The choline-deficient L-amino acid-defined (CDAA) diet based NASH model is known to induce hepatomegaly, hepatic steatosis and triacylglycerol accumulation because of the impaired liver lipid secretory capacity during the CDAA diet^18^. Recently, the CDAA diet supplemented with different cholesterol concentrations has been evaluated in Wistar rats^19^. Liver inflammation markedly increased in CDAA animals throughout all time points indicated by mRNA markers and immune cell infiltration. Notably, the cholesterol supplementation increased the lipotrope properties of the CDAA diet and further promoted a fibrotic phenotype. Among the cholesterol supplementations tested, 1% cholesterol showed the most suitable phenotype for pharmacological testing^19^.

For the present study, we used mRNA sequencing of liver samples in combination with LC-MS based proteomics of liver and plasma samples from the CDAA + 1% cholesterol model for preclinical biomarker discovery. We compared our transcriptomic data to public human NASH data to show the relevance of the induced changes for the human disease. We observed good correlation between transcript and protein expression for the majority of regulated genes. Furthermore, we could detect some of these changes also in the plasma. Ranking by multi-dimensional attributes derived from our data and prior biomarker evidence revealed known biomarker candidate proteins. In addition, we identified several candidates without prior NASH biomarker evidence. In summary, the present study provides a comprehensive multi-omics framework for preclinical NASH biomarker discovery. Moreover, it shows the utility of different omics technologies for this approach, which is adequately applicable in clinical settings.

## Results

### RNA-Seq reveals strong gene expression changes relevant for the NASH phenotype

**Figure 1:**
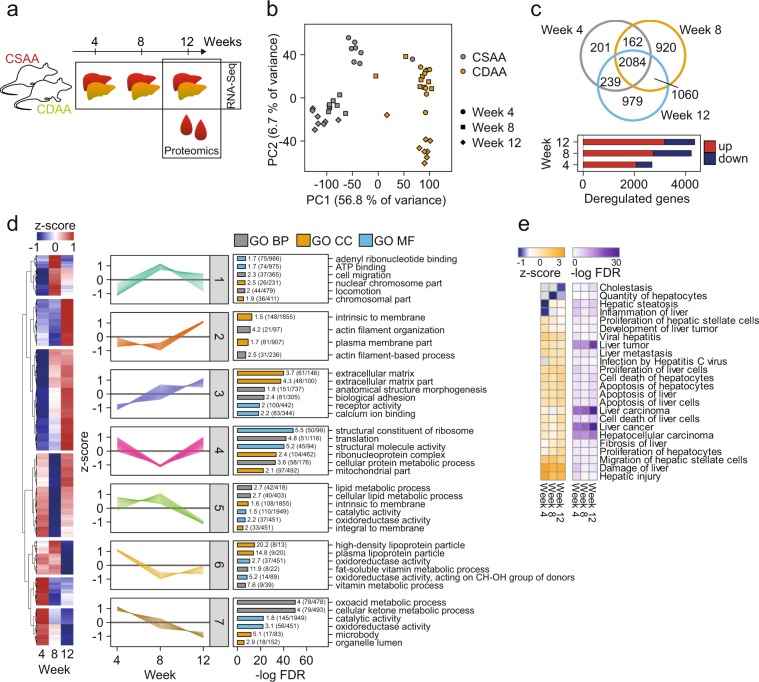
Transcriptomic characterization of the rat CDAA model. (**a**) Overview of experimental layout for multi-omics model characterization. (**b**) Principal component analysis scores plot of RNA-Seq data from liver of weeks 4, 8, and 12 of CSAA and CDAA diet. (**c**) Number of deregulated genes (FC > |1|, Benjamini-Hochberg adj. *p* value < 0.01) at different time points as bar diagram and Venn diagram. (**d**) Hierarchical clustering of *z*-scored gene expression ratio time profiles. Overrepresentation analysis of Gene Ontology (GO) terms Biological Process (BP), Cellular Component (CC), and Molecular Function (MF) in clusters was done using Fisher’s exact test (Benjamini-Hochberg adj. *p* value). Shown here are the two most significant categories (category size <2000 genes, enrichment factor >1, intersection size >7 genes). Supplementary Table 1 contains the full result table. (**e**) Hepatotoxicity functional overrepresentation analysis from IPA for comparison of different time points (Benjamini-Hochberg adj. *p* value < 0.01, *z*-score > |0.75|).

Recently, we investigated the CDAA diet with different supplementary combinations using Wistar rats for their suitability as a preclinical NASH model^19^. From this experiment we selected the CDAA diet supplemented with 1% cholesterol (in the following abbreviated as CDAA) for molecular profiling because it shows the most relevant phenotype. To gain insight into molecular mechanisms of disease progression we analyzed liver tissue from diseased CDAA and choline-supplemented L-amino acid-defined (CSAA) control animals at 4, 8, and 12 weeks by RNA-Seq (Fig. 1a).

RNA integrity of all samples was very good (RIN > 8). Samples were sequenced at a sequencing depth of 20 to 30 million single end reads per sample with a high rate of >70% exonic reads that mapped uniquely to the rat genome. Sample-to-sample correlation based on log_2_ normalized read counts was very high within each group (*r* Pearson > 0.95).

Unsupervised principal component analysis (PCA) revealed a clustering of sample groups, except for three outlier animals (Fig. 1b). The first principal component (PC1) separated samples from CDAA and CSAA diet. PC1 values of CDAA samples were generally negative with further decreasing values with the duration of the CDAA diet (whereby samples from CDAA diet after week 8 and 12 are relatively close to each other). In PC2, samples from both conditions clustered with respect to the duration of the experiment, confirming the necessity of having time matched controls. However, this effect seems to be small compared to the diet effect as indicated by the explained variance (<7% in PC2 compared to >56% in PC1).

The clear difference between CDAA and CSAA diet was also reflected in the differential gene expression analysis (adj. *p* value < 1%, log_2_ FC > |1|, Fig. 1c, bars). The majority of genes at each time point were upregulated, increasing from 2000 to more than 3000 from week 4 to week 12. Despite differential gene expression increased over time, a subset of 2084 genes was consistently regulated at all three time points (Fig. 1c, Venn diagram). The total number of time point specific differentially expressed genes increased from 7% at week 4 to 22% in week 8 and week 12. In addition, the specific overlap was highest between 8 and 12 weeks at approximately 20%.

To gain insight into regulated pathways and associated molecular functions among all differentially expressed genes (n = 5645) we used hierarchical clustering to group co-regulated genes across the time course into seven different clusters. We tested for overrepresentation of GO terms using Fisher’s exact test focusing on the top two significant hits for each term (Fig. 1d, Supplementary Table S1). Of note, genes with a consistent linear increase (Cluster 3) showed a highly significant overrepresentation of ECM and collagen genes corroborating the establishment of pro-fibrotic processes in the liver over time. Cellular components of chromosomes and biological process of cell migration were significantly overrepresented in Cluster 1 that showed a transient pattern of gene expression with an increase from 4 to 8 weeks and a decreasing expression from 8 to 12 weeks. Cluster 6 and Cluster 7, which show a decreasing expression pattern over time, revealed a significant overrepresentation of cellular components of functional hepatocytes like lipoproteins and metabolic enzymes. This is in line with the decrease of hepatocyte function and lipid secretory capacity between 4 and 8 weeks described for CDAA diet fed rats^18^. Interestingly, lipid metabolism related processes (lipid biosynthetic process, fatty acid metabolic process) were also enriched in Cluster 5, where the decrease in expression started between 8 and 12 weeks. Lipid accumulation in the liver of CDAA fed rats was shown to start much earlier than 8 weeks^19^, and lipid export continued to decrease from 4 to 8 weeks (Cluster 6). This may indicate a buffering capacity in the liver that only after 12 weeks leads to coordinated downregulation of lipid metabolism genes.

We performed an additional functional assessment of the observed gene signatures using hepatotoxicity annotations within Ingenuity Pathway Analysis (IPA) (Fig. 1e). Accordingly, the time course from four to 12 weeks revealed significant overrepresentation of functions of liver fibrosis, inflammation, and apoptosis. Strikingly, we observed a clear increase in *z-*scores of hepatic steatosis and inflammation from week four though week 12. Taken together, we provide an in depth characterization of gene expression in CDAA fed Wistar rats. The data reflected molecular mechanisms of diet induced lipid accumulation and metabolism in the liver including inflammatory and fibrotic pathways.

### Proteomics of liver tissue and plasma corroborate findings from gene expression

Protein expression is regulated on different levels and hence readout of protein encoding RNA abundance can be misleading e.g. in non-steady state conditions. We therefore studied proteomic changes in samples with established fibrosis at week 12 of liver tissue and plasma. We used tandem-mass-tags (TMT) with nano-LC-MS/MS to relatively quantify 3273 and 433 proteins in liver and plasma with a protein false discovery rate (FDR) of <1%, respectively. Overall 2348 and 163 proteins changed significantly in liver and plasma, respectively (Student’s T-test, permutation based FDR < 1%). The majority of regulated proteins were upregulated in both analyzed matrices, with an almost identical ratio of the number of up- versus downregulated proteins (3.2 for liver, 3.3 for plasma). This indicates that perturbations in the liver primarily drove changes observed in plasma.

**Figure 2:**
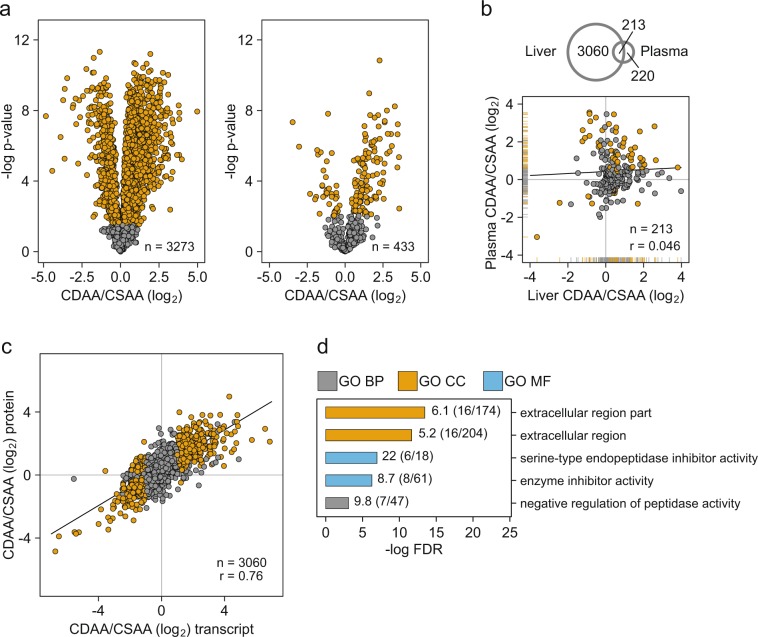
Proteomics analysis of the CDAA model at 12 weeks and comparison to transcriptomic data. (**a**) Volcano plots of protein changes observed in liver (left) and plasma (right). Significant changes are colored (T-test, permutation based FDR < 1%). (**b**) Venn diagram showing the overlap of liver and plasma proteomics data. Lower plot: corresponding log_2_ fold changes of liver and plasma proteins. Significantly changing proteins in both matrices are colored. (**c**) Correlation of individual transcript to protein log_2_ fold changes at week 12. Significant changes on both levels are colored. (**d**) Top two overrepresented sets of anti-regulated features on transcript and protein level (n = 49, Benjamini-Hochberg adj. *p* value < 0.05). Statistical overrepresentation of Gene Ontology (GO) terms (GO Database released 2019-01-01) for Molecular Function (GO MF), Cellular Component (GO CC), and Biological Process (GO BP) was tested against all overlapping features as reference list with the Panther online tool^68^ (http://pantherdb.org/).

Changes in protein expression between healthy and diseased liver tissue ranged widely from −5 to 5 (log_2_) (Fig. 2a). Among the highest upregulated proteins, we found ECM proteins like EFEMP1 and LTBP1. Interestingly, the strongest downregulated proteins included several drug metabolizing enzymes (CYP2C9, CYCP2C8, AOX3) and proteins involved in cellular amino acid catabolism such as GLS and HAL. In line, oxidoreductase activity related genes representing cytochrome P450 enzymes were significantly overrepresented in transcriptomics clusters that showed decreasing tendency (Fig. 1d, Clusters 5 and 6). Hence, drug-metabolizing capacity of CDAA livers seemed to be strongly compromised. Drug metabolizing enzyme expression was previously studied in human NASH showing strong regulation of cytochrome P450 enzymes^20^, and transporters^21^. This is a highly relevant finding for drug discovery and development because profound differential expression of drug metabolizing enzymes and transporters may affect the pharmacokinetics of drug candidates and therefore complicate drug discovery programs.

As expected, we observed less pronounced protein changes in plasma compared to the liver. However, 38% of the detected plasma proteins changed significantly after 12 weeks CDAA diet (Fig. 2a). Strikingly, among the top 10 downregulated plasma proteins we found 4 apolipoproteins (APOC2, APON, APOA2, APOC4) in line with molecular mechanisms of the CDAA diet leading to liver malfunctioning via the impaired lipid export from the liver^18^.

A comparison of protein log_2_-fold changes between liver and plasma showed no correlation between the two (n = 213, *r* Pearson = 0.05, Fig. 2b). However, there was a higher number of proteins with a co-regulation in liver and plasma than with an opposite regulation pattern. Moreover, the top co-regulated proteins correspond to secreted matrix proteins which represent a set of attractive biomarker candidates including COL6A2, ADAMTSL2, and LGALS3BP (see section Potential NASH biomarkers).

To compare our transcriptomics and proteomics readouts in liver tissue at 12 weeks we analyzed log_2_-ratios of liver transcripts and proteins together. We observed a strong co-regulation of transcripts and proteins with 3060 overlapping pairs (*r* Pearson = 0.76, Fig. 2c). This indicates that transcriptional regulation is the major driver of the observed molecular changes in the CDAA vs. CSAA diet, which also translates to protein regulation. However, we discovered also 49 anti-regulated transcript-protein pairs with significantly decreased RNA levels, but increased protein levels. To learn more about the anti-regulated proteins we performed an overrepresentation analysis of Gene Ontology terms for Molecular Function, Cellular Component, and Biological Process as described in the figure legend (Fig. 2d). We found a significant overrepresentation of extracellular proteins, as well as a strong signal for peptidase inhibitors (Fig. 2d).

### Key disease processes in CDAA rats are relevant for NASH in human

**Figure 3:**
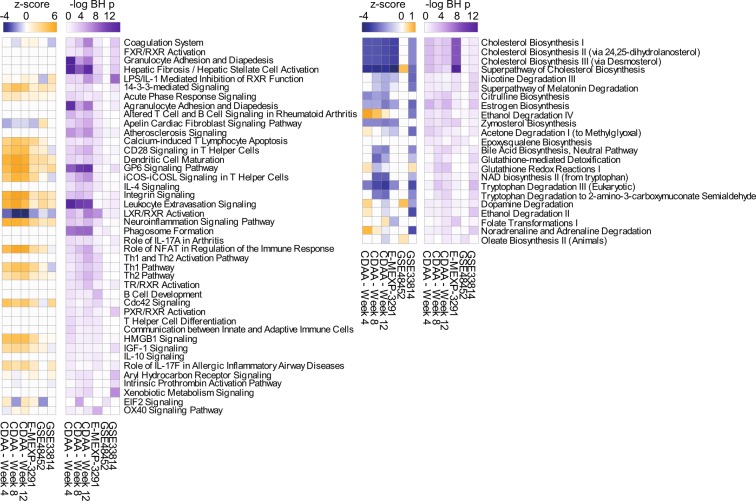
Comparison of rat CDAA to human NASH mRNA datasets on pathway level. Analysis match of overrepresentation analyses of canonical pathways using IPA of all rat data and three selected human datasets. Data are filtered to give at least one hit in a human study and one rat CDAA time point with Benjamini-Hochberg adj. *p* value < 0.05. Left panel: Signaling pathways, right panel: metabolic pathways (see Supplementary Table S2 for details). *Note*: Due to different reference datasets, the *p* values of the analyses are not directly comparable.

To put the CDAA model into context with human disease we compared our transcriptomics data to publicly available gene expression studies from NASH patients using IPA (Array express E-MEXP-3291^21^, Gene Expression Omnibus GSE48452^22^ and GSE33814^23^). Overall, we found 65 pathways significantly regulated in at least one human and one rat CDAA sample (Benjamini-Hochberg adj. *p* value < 0.05, Fig. 3; see Supplementary Table S2 for details). The majority of overrepresented pathways correspond to signaling pathways. Based on *z*-score, most of them are activated in the disease (e.g. hepatic stellate cell activation and leucocyte extravasation) whereas LXR/RXR activation is suppressed. The most enriched metabolic pathways were Cholesterol biosynthesis, Glutathione-mediated detoxification and Tryptophan degradation. All three are also consistently suppressed in human and in CDAA rats.

Although there is a general agreement of activated and deactivated pathways across the investigated data sets, we observed considerable variability in between the human datasets and lower evidence levels of the human data vs. the rat NASH model. For example, there was no single significantly overrepresented pathway common to all three investigated human datasets. In summary, significances of canonical pathways did not overlay widely. Nevertheless, the tendency of regulation, as measured by the *z*-score, was overlapping in almost all cases highlighting similar mechanistic changes in the rat model and human disease.

### Potential NASH biomarkers derived from CDAA model

As described above, pathological changes in the liver of diseased animals go along with massive changes of transcript and protein expression. Many of the strongest regulated transcripts and proteins showed a high positive or negative correlation with liver fibrosis. Consequently, these molecular signatures provide alternative disease and efficacy biomarkers as already shown by TaqMan analysis for the pro-fibrotic markers *Col1a1, Acta*2 and *Ctgf*^19^. However, our aim was to identify potential *in vivo* plasma biomarkers suitable for longitudinal preclinical studies and predictive for the degree of liver fibrosis. Consequently, we assumed these plasma biomarkers should correlate to the liver phenotype as assessed by histology and molecular features, *i.e*. liver RNA and protein expression. Therefore, we integrated the different readouts using a weighted sum model and ranked those plasma biomarkers high that showed a strong correlation to the disease state of the liver.

**Figure 4:**
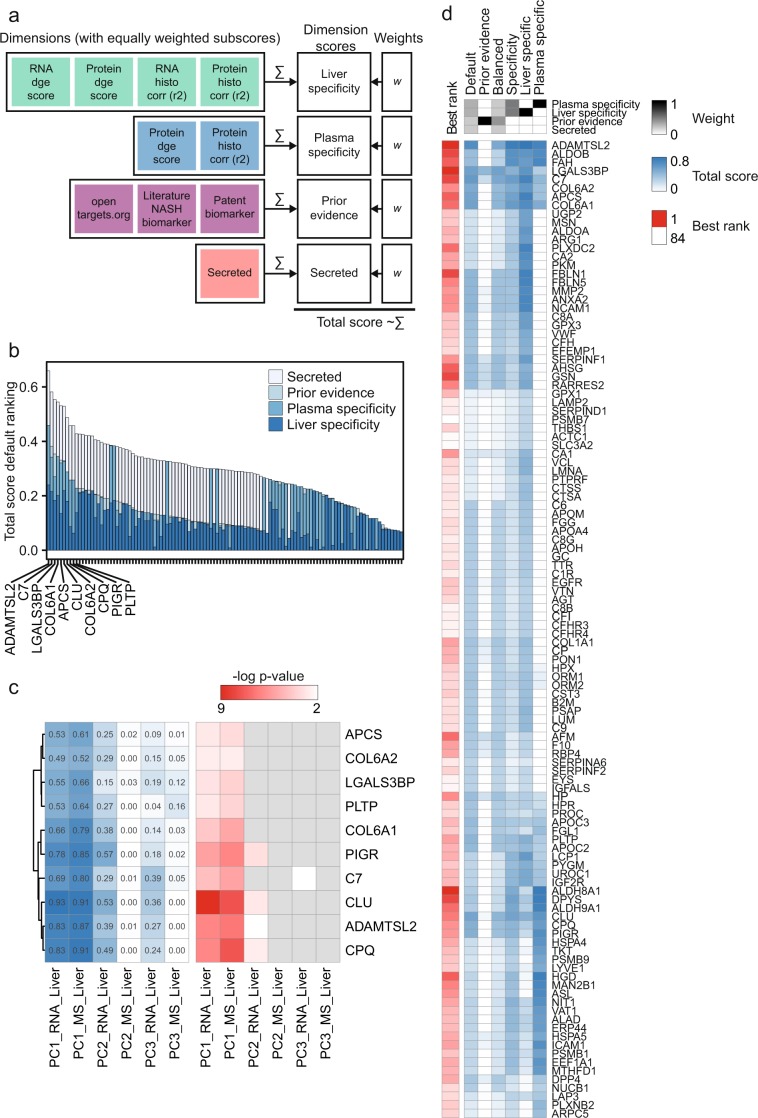
Multi-dimensional attribute ranking for biomarker discovery. (**a**) Biomarker scoring scheme using the weighted sum of multiple normalized subscores (see Methods for description, Supplementary Table S3). (**b**) Ranking by total biomarker score using the default weight setting with the contribution of each subscore. The top 10 biomarkers are labeled by coding gene name. (**c**) Linear regression analysis of the top 10 ranked biomarkers (see b) using protein plasma intensity as predictor (x) for the first three components of the corresponding sample in the liver RNA and protein PCAs. PC1, which separates CDAA vs. CSAA samples, showed the best correlation to protein plasma intensity. Left heatmap: *r*^2^ values of regression analysis, right heatmap: *p* values of correlation. *p* values > 0.01 were shaded in grey. Clustering by *r*^2^ (**d**) Clustering of total scores obtained from the sensitivity analysis using six different weight settings. The corresponding weight settings are displayed at the top (grey-scale heatmap). The best rank for each protein in any of the weight settings is shown on the left (log_2_ of rank).

In a first step, we selected only rat proteins that have a corresponding human homolog and were detected in liver and plasma (see Methods, and Supplementary Table S3). We then generated a scoring table with four dimensions. For the first two dimensions, we connected the three omics data sets via linear correlation between expression signal and liver fibrosis (fibrosis score retrieved from^19^). Thus, we obtained subscores for liver RNA, liver protein and plasma protein specificity. Second, we prioritized secreted proteins as major class of potential biomarker proteins because they were highly enriched in our initial subset of detected plasma proteins (see Supplementary Table S3). We complemented this data by prior biomarker evidence for each gene and protein compiled from published data as an additional dimension (Table 1). These multi-dimensional attributes were combined by using a staggered weighted sum approach (Fig. 4a): First, we mapped all considered subscores to the four major dimensions (plasma specificity, liver specificity, prior biomarker evidence, secreted protein). Secondly, we calculated each dimensional score as the sum of its subscores. Finally, we calculated the total biomarker score as the weighted sum of the dimensional scores (see Fig. 4a). Figure 4b shows the *a priori* defined weights for the final ranking with a strong bias on our experimental data (“default”, plasma 0.3, liver 0.3, prior evidence 0.2, and secreted protein class 0.2). The top 10 predicted biomarkers are listed in Table 2. The plasma levels of these candidates correlated well with the first component of PCA derived from the liver omics data indicating that they are suitable classifier for CDAA *vs*. CSAA liver samples (Fig. 4c). The linear regression models had a Pearson correlation coefficient of *r*^*2*^ > 0.49 and *p* < 0.01 for all proteins among the top 10 while the best performing candidate *Clu* had *r*^2^ > 0.92 and *p* < 10^−7^. Further, to validate the predicted biomarkers, we have compiled information on protein function and assessed tissue specificity by RNA expression profiling. The RNA tissue distribution strengthens the link between plasma protein detection and biomarker tissue specificity (see Table 2).

**Table 1:** Multi-dimensional ranking dimensions and their subscores.

Dimension	Subscore (equally weighted)
Plasma specificity	Plasma Protein Differential Expression (dge score)
	Plasma Protein - Histo Correlation (r^2^)
Liver Specificity	Liver Protein Differential Expression (dge score)
	Liver RNA Differential Expression (dge score)
	Liver Protein - Histo Correlation (r^2^)
	Liver RNA - Histo Correlation (r^2^)
Prior Evidence	Association to Fibrosis (OpenTargets overall score)
	Literature NASH biomarker (GeneRifs observed vs expected score)
	Literature NASH biomarker (Pubmed observed vs expected score)
	Patent NASH biomarker Somalogic (present = 1, 0 otherwise)
	NASH biomarker Integrity/MetaCore (present = 1, 0 otherwise)
Protein Class	Secreted (secreted = 1, 0 otherwise)

All proteins were scored according to four different dimensions using the sum of equally weighted subscores.

**Table 2:** Top 10 biomarker candidates with annotated protein function, sign of regulation in liver on RNA level (LR), in liver on protein level (LP), and in plasma on protein level (PP) after 12 weeks of CDAA vs. CSAA diet.

Gene Name	Protein Function (UniProt/Swissprot)	Regulation LR, LP, PP	Top2 enriched tissues (median fold change)
ADAMTSL2	#N/A	Up, Up, Up	Adrenal Gland (5.6), Kidney (4.4)
C7	Constituent of the membrane attack complex (MAC) that plays a key role in the innate and adaptive immune response by forming pores in the plasma membrane of target cells.	Up, Up, Up	Adrenal Gland (7.6), Ovary (3.2)
LGALS3BP	Promotes integrin-mediated cell adhesion.	Up, Up, Up	HSC - TGFb 2.5 ng (4.7), Stomach (2.9)
COL6A1	Collagen VI acts as a cell-binding protein.	Up, Up, Up	HSC - TGFb 2.5 ng (11.1), HSC - Control (8.8)
APCS	Can interact with DNA and histones and may scavenge nuclear material released from damaged circulating cells.	Down, Down, Down	Liver (25.7), Gall Bladder (1.3)
CLU	Isoform 1 functions as extracellular chaperone that prevents aggregation of nonnative proteins.	NR,Up, Up	Cerebral Cortex (4.7), Liver (3.6)
COL6A2	Collagen VI acts as a cell-binding protein.	Up, Up, Up	HSC - TGFb 2.5 ng (8.9), HSC - Control (7.3)
CPQ	Carboxypeptidase that may play an important role in the hydrolysis of circulating peptides.	Down, NR, Down	Thyroid Gland (6.8), Gall Bladder (2.0)
PIGR	This receptor binds polymeric IgA and IgM at the basolateral surface of epithelial cells.	Down, NR, Up	Duodenum (8.7), Colon (5.7)
PLTP	Facilitates the transfer of a spectrum of different lipid molecules […]. Essential for the transfer of excess surface lipids from triglyceride-rich lipoproteins to HDL, thereby facilitating the formation of smaller lipoprotein remnants, contributing to the formation of LDL, and assisting in the maturation of HDL particles. PLTP also plays a key role in the uptake of cholesterol from peripheral cells and tissues that is subsequently transported to the liver for degradation and excretion. Two distinct forms of PLTP exist in plasma: an active form that can transfer PC from phospholipid vesicles to high-density lipoproteins (HDL), and an inactive form that lacks this capability.	Up, Up, Up	Placenta (7.2), Gall Bladder (2.5)

The column on the left shows the tissue specificity for each gene in a panel of RNA-Seq data from 27 normal tissues (ArrayExpress E-MTAB-1733) complemented by RNA-Seq data from stellate cells, *i.e*. the control group and a sample group treated with TGFb (Gene Expression Omnibus GSE78853). Enrichment factors correspond to the fold change of the median expression in enriched tissue vs. median of median across all normal tissues.

We tested the sensitivity of the biomarker ranking to the *a priori* set weights by applying five additional weight settings (Table 3). Figure 4d shows the total scores for each protein obtained from the different weight settings as a heatmap. Strikingly, all rankings including experimental data revealed a consistent block of highly ranked proteins. In contrast, prior knowledge based ranking that did not consider experimental data from the present study, prioritized different top biomarker candidates. This suggests that the selection of the best soluble plasma biomarker candidates for the investigated disease setting requires suitable experimental data.

**Table 3:** Weight sets of six different tested rankings.

#	Dimension *(columns)* Ranking *(rows)*	Plasma Specificity	Liver Specificity	Prior Evidence	Secreted Protein Class
1	Default	0.3	0.3	0.2	0.2
2	Prior Knowledge	0.0	0.0	1.0	0.0
3	Balanced	0.2	0.2	0.4	0.2
4	Liver/Plasma specific	0.5	0.5	0.0	0.0
5	Liver specific	0.0	1.0	0.0	0.0
6	Plasma specific	1.0	0.0	0.0	0.0

The individual rankings were done on the total scores obtained by the weighted sum of individual dimension scores. For each ranking, all weights listed in Table 3 sum up to 1.

In summary, we found a number of markers in plasma that highly correlate to the liver phenotypes of the rat CDAA NASH model. Some candidates (e.g. ADAMTSL2, CPQ, see discussion) are unprecedented in this context and therefore represent promising new candidates which will be investigated in the future. Others have been previously proposed as biomarkers for human liver diseases including NASH, validating our approach. Additionally this underlines the translatable nature of the rat CDAA model.

## Discussion

NASH is a chronic liver disease affecting a large part of the global population with an alarming increase in prevalence^24^. The therapeutic need is high since there is currently no approved drug available^4^. Non-invasive plasma biomarkers, both for preclinical research and for clinical development, could accelerate the development of new drugs. To our knowledge, we present for the first time a deep molecular characterization of disease progression in a rat animal NASH model based on transcriptomics complemented by MS-based proteomics of liver and plasma. We show that expression of certain genes and proteins correlated to liver histology and translated into robust detectable plasma protein changes. These proteins represent biomarker candidates, some of which have been described before in the context of NASH and fibrosis biomarker research, but some are novel.

Our in depth molecular characterization of the rat CDAA model by deep sequencing revealed specific molecular signatures that could be functionally linked to previously described disease phenotypes i.e. steatosis, immune cell infiltration and fibrosis^19^. We found decreased levels of apolipoproteins in plasma and decreased expression of genes involved in lipoprotein particles in line with known physiological effects of choline deficiency causing lipid accumulation in the liver. Moreover, ECM related genes showed a continuous expression increase. Analysis of hepatotoxicity functions in IPA revealed significant overrepresentation of genes involved in liver fibrosis, steatosis, apoptosis, as well as stellate cell proliferation. Together, using RNA-Seq combined with hierarchical clustering and IPA overrepresentation analysis, we revealed time dependent changes manifesting the phenotypic changes observed in this model.

Our proteomics data correlated very well to the transcriptomics data from the same liver samples based on differential expression statistics (Fig. 2c). This indicates a strong transcriptional regulation of gene expression potentially due to the harsh conditions upon choline deficiency inducing massive changes in the liver. Of note, a similar observation has recently been made for a methionine and choline-deficient diet induced mouse model comparing transcriptomics and proteomics read-outs^25^.

We show that molecular changes found in the investigated NASH model recapitulate major features of human NASH (Figs. 1 and 3). The time point dependent gene expression changes as reflected in the enriched canonical pathways of CDAA rats mainly coincided with those observed in human (Fig. 3). The analyzed human data showed generally a high variability indicating a need for additional human data with increased statistical power (i.e. larger sample size and stratification with respect to fibrosis stage) and the application of state-of-the-art transcriptomic analysis by next generation sequencing. However, there was a reasonable agreement between our rat data and the human data. Among metabolic pathways, the strong downregulation of cholesterol related pathways is also observed in NASH mouse models using high levels of dietary cholesterol^17,26^. The dietary cholesterol concentrations in those models is used to mimic the human metabolism^17,27,28^. High cholesterol diets in the context of a NASH mouse model was suggested to affect fatty acid beta-oxidation, liver VLDL secretion and neutral bile acid synthesis^28^.

To discover biomarker candidates from detectable plasma proteins we developed a multi-attribute ranking approach. The majority of high-ranked candidates was significantly upregulated by CDAA diet and positively correlated to liver fibrosis, hence they are probably related to pro-fibrotic processes. Therefore, the source of these proteins is presumably the ECM (e.g. ADMATSL2, LGALS3BP, Clusterin, COL6A1, and COL6A2). Strikingly, five out of the top 10 biomarker candidates show a high liver tissue or stellate cell type specificity (liver: APCS, CLU; hepatic stellate cells: COL6A1, COL6A2, LGALS3BP).

Among the well-known top candidates, there is Galectin-3-binding protein (LGALS3BP), a secreted protein with high biomarker evidence. LGALS3BP is a glycosylated, excreted protein that binds other ECM proteins (collagens, fibronectin) and promotes cellular adhesion^29^. It has been proposed as biomarker for severe NASH before^30–32^ and recently for NAFLD and liver cirrhosis^12^. In addition, it was postulated as disease severity and treatment efficacy biomarker for liver fibrosis in Hepatitis B and C^33–35^. Polymeric immunoglobulin receptor (PIGR) is an IgA and IgM transporter linking adaptive and innate immunity^36^. PIGR induces epithelial mesenchymal transition through the activation of SMAD signaling^37^. It was identified as potential biomarker for liver metastasis from colorectal cancer^38^. PIGR levels were also found elevated in human plasma of NAFLD and cirrhotic patients^12^, again corroborating our findings from the CDAA rat model. Clusterin (Apolipoprotein J, encoded by CLU gene) is a secreted, glycosylated protein with chaperone activity that helps stabilizing non-native extracellular proteins in an ATP independent manner^39^. Thus, Clusterin is probably involved in extracellular protein aggregation and clearance thereof. In addition, its expression is increased by cellular stress^40^. The protein has been discussed as a potential serum biomarker for hepatitis B (HBV) and C virus (HCV) induced liver fibrosis. In contrast to our study showing an upregulation in disease conditions in liver and plasma, it was negatively correlated with liver fibrosis severity in human HBV and HCV^12,41,42^. Interestingly, plasma Clusterin increased also in an LDLR^−/−^ mouse steatosis model under high fat diet, and obese human subjects^43^. Apart from fibrosis, Clusterin serves as a safety biomarker for kidney function (see https://www.fda.gov). Amyloid P component, serum (APCS, also known as PTX2 or SAP) has immune regulatory functions, whose tissue expression is highly specific for the liver. Intriguingly, it was shown to inhibit monocyte to fibrocyte differentiation^44^. APCS is even proposed as a therapeutic for fibrosing diseases^45^. Consistent with our results, APCS levels decreased in plasma of a miniature swine NASH model^46^ and human NASH patients^13^. Conversely, it was strongly increased in a mouse model of high fat diet induced NAFLD^12^, indicating potential specificity for disease stage or cause of fibrosis.

Among our top candidates, we also found proteins that lack prior biomarker evidence for liver related diseases. Carboxypeptidase Q (CPQ) is involved in thyroid hormone activation^47^ and its expression increased in regenerating liver^48^. However, its involvement in liver fibrosis or NASH remains unknown. Another candidate is ADAMTS-like 2 (ADAMTSL2), an atypical member of the ADAMTS family of proteins, which lacks metalloprotease and disintegrin-like domains^49^. The molecular function of ADAMTSTL2 is mostly unknown to date. Mutations in the ADAMTSL2 gene cause geleophysic dysplasia 1 (GPHYSD1) [MIM:231050], with symptoms of hepatomegaly^50^. ADAMTSL2 interacts with ECM modulator proteins including LTBP1, FBN, and members of the LOX family^50–52^. Of note, individuals with geleophysic dysplasia have higher total and active levels of TGFb^50^. ADAMTSL2 represents an unprecedented biomarker candidate for NASH or fibrosis although existing evidence suggests its involvement in ECM biology.

Our approach presented here also has limitations, specifically with regard to human translatability and NASH sub-type classification. Human NASH is a complex disease with different pathological processes involved (i.e. steatosis, hepatocyte dysfunction, inflammation and fibrosis). Consequently, it will be difficult to predict a particular stage or sub-type of NASH just from a single plasma biomarker. Therefore, a combination of markers or marker signatures will be finally more predictive and informative for clinical applications. However, this will also require larger data sets with a higher variability across affected individuals. Nevertheless, the molecular functions of the top 10 biomarker candidates (Table 2) are linked to immune response (PIGR, C7, APCS), lipid metabolism (PLTP), and ECM (ADAMTSL2, COL6A1, COL6A2, LGALS3BP, CLU) and all relevant to NASH pathology.

We have used a proteomics approach to assess the protein levels after depletion of abundant plasma proteins. However, due to the huge dynamic range of plasma proteins we still only detected a subset of proteins. Hence, we possibly have missed interesting biomarkers that remain in the lower abundant fraction^53^. It is possible to increase depth in plasma protein analysis with MS-based proteomics using higher effort (*i.e*. more extensive depletion and fractionation) or other methods, like the use of modified aptamers^54^. However, for the present study we have decided for an approach that is untargeted and sensitive enough to analyze a reasonable number of plasma proteins.

So far, we did not validate our findings in a second study nor did we evaluate the observed plasma protein patterns in another NASH model. Furthermore, the clinical relevance of the biomarker candidates is limited since preclinical disease models only partially reflect the human situation. However, preclinical biomarkers considerably support NASH drug development by allowing a better study design (rationale for duration of the experiment, increased statistical power due to group randomization before drug treatment). However, the further qualification and development of biomarker candidates require additional validation that could directly be done with human samples.

In summary, we show here a methodical setup by using multi-omics data with classical liver histopathology. We show its feasibility and power in preclinical settings, which suggests that it is also straightforwardly applicable to human samples. Although technically challenging, it is possible to get enough liver tissue from biopsies for histopathology, mRNA and protein preparation, while collection of plasma samples should not be a technical limitation. We identified several markers of related human liver diseases underpinning the suitability of our approach in preclinical models and translatability of the CDAA rat model. Thus, our analyses have the potential to advance the development of biomarkers for preclinical and clinical trials and furthermore support the understanding of the pathophysiology of NASH.

## Methods

### Animal study

The detailed description of the performed animal study is given in^19^. Briefly, male Wistar rats (RjHan:WI, 6 weeks of age, 200–250 g, Janvier Labs, Le Genest-Saint-Isle, France) were acclimatized in 12 h/12 h light/dark cycle in pairwise housing. To induce a NASH phenotype groups of eight animals were fed a choline-deficient l-amino acid-defined diet (kcal %; protein 11%, fat 31%, carbohydrates 58%) supplemented with 1% cholesterol (E15666-94) or a choline-supplemented l-amino acid-defined control diet (E15668-04; kcal %; protein 12%, fat 16%, carbohydrates 72%) for 4, 8, or 12 weeks. All diets were obtained from ssnif Spezialdiäten GmbH (Soest, Germany).

### Ethical statement

The animal experiment was conducted in accordance with the German Law on the Protection of Animals and performed in accordance with EU guidelines for the accommodation and care of animals used for experimental and other scientific purposes. The experiment was approved under the license number 13-011-G by the official regional council Tuebingen, Germany and is in detail described in^19^.

### Transcriptomics

#### RNA isolation

RNA was isolated and prepared as described in detail in^19^. In brief, pieces of 50 mg of liver tissue were homogenized in RNAeasy lysis buffer (Qiagen, Hilden, Germany) with 1% 2-mercaptoethanol. Total RNA was then isolated according to the manufacturer’s protocol (RNeasy, Qiagen, Hilden, Germany).

RNA concentrations and purity have been determined using a NanoDrop ND-1000 UV–Vis Spectrophotometer (Thermo Scientific, Karlsruhe, Germany) at 260 nm and 260/280 nm ratio, respectively. All samples were stored at −80 °C before further analysis by RNA-Seq.

#### Illumina library preparation and sequencing

For library preparation, RNA quality and concentration was measured using the Fragment Analyzer from AATI (now Agilent) with the total RNA Standard Sensitivity protocol. The Sequencing library was prepared from 200 ng total RNA with the TruSeq® Stranded mRNA LT-Set B (RS-122-2102, Illumina Inc., San Diego, CA) producing a 275 bp fragment including adapters in average size. In the final step before sequencing, seven individual libraries were normalized and pooled together using the adapter indices supplied by the manufacturer. Pooled libraries have been clustered on the cBot Instrument from Illumina using the HiSeq® 3000 GD-410-1001 3000/4000 SR Cluster Kit (Illumina Inc., San Diego, CA). Sequencing was performed as 85 bp single-end reads and 7 bases index read on an Illumina HiSeq. 3000 instrument at a sequencing depth of approximately 60 million reads per sample using FC-410-1001, HiSeq® 3000/4000 SBS Kit (50 cycles, Illumina Inc., San Diego, CA).

### Proteomics

#### Sample preparation for TMT based proteomics

30–50 mg of liver samples were homogenized in Pierce™ IP lysis buffer (1:10 w/w) and 1x Halt™ Protease Inhibitor Cocktail (both Thermo Fisher Scientific) using a Precellys® Evolution homogenizer (Bertin). Protein concentration was determined from the homogenate supernatants using the Bradford assay (Sigma-Aldrich). For plasma samples, the six most abundant proteins were depleted using the Seppro® rat Spin Column (Sigma-Aldrich) before determining protein concentration with the Bradford assay. 100 µg of protein (on average 12 µL lysate) were denatured with 1% SDS and reduced with 2 µL 0.5 M tris(2-carboxyethyl)phosphin for 1 hour at 55 °C. Cysteine alkylation was performed by adding 5 µL of 375 mM iodoacetamide and incubated for 30 min at room temperature in the dark. To precipitate proteins, 600 µL of cold acetone were added and incubated for 1 hour at −20 °C. The samples were centrifuged for 10 min at 16.000 rpm and the pellet was washed with additional 65 µL of chilled acetone. On-pellet digest was done in 100 µL 100 mM triethylammonium bicarbonate and trypsin/LysC mix (Promega) at a 1:25 enzyme to protein ratio over night at 37 °C.

TMT labelling was performed following the manufacturer’s instructions (Thermo Fisher Scientific). We multiplexed the 16 samples into two TMT-8-plexes for the liver and one TMT-6-plex and one TMT-10-plex for the plasma distributing healthy and diseased animals equally between TMT-plexes. We fractionated peptides prior to LC-MS analysis into eight fractions using the Pierce™ High pH Reversed-Phase Peptide Fractionation Kit (Thermo Fisher Scientific) according to the manufacturer’s instructions.

#### Nano-LC-MSMS of TMT labelled peptides

Samples were analyzed with an UltiMate 3000 RSLCnano LC system coupled to an Orbitrap Fusion Lumos Tribrid mass spectrometer (Thermo Fisher Scientific). Per fraction, 2 µg of labelled peptides (corresponding to volumes of 16 µL for liver samples and 6 µL for plasma samples) were first trapped on a PepMap100 C18, 5 × 0.3 mm, 5 µm pre-column (3% acetonitrile, 0.1% formic acid). Peptides were then separated on an EASY-Spray C18, 75 cm × 75 µm, 2 µm column (Thermo Fisher Scientific) heated to 50 °C at 300 nL/min using a gradient of 3–28% eluent B (80% acetonitrile, 0.1% formic acid) in 210 min and 28–40% eluent B in 30 min followed by a 10 min 95% eluent B wash step and 1 hour re-equilibration.

The mass spectrometer was operated with the multi-notch synchronous precursor selection (SPS) mode. Precursor spectra were acquired from 375–1500 m/z at 120,000 resolution with an AGC target of 4*10e5 and maximum injection time of 50 msec. The top five precursor ions were isolated with a 0.7 m/z window and fragmented by 35% CID. MS2 scans were acquired in the ion trap in turbo ion mode, 1*10e4 AGC target and 50 msec maximum injection time. Dynamic exclusion was set to 60 sec. For the MS3 spectra, the top five MS2 fragments were fragmented by HCD at 65% and acquired in the Orbitrap at 60.000 resolution, 1*10e5 AGC target and 150 msec maximum injection time. Ions were not accumulated for all parallelizable time.

### Data analysis

#### mRNA-Seq analysis

mRNA-Seq data analysis was performed as previously described^55^. We aligned sequenced reads to the rat genome (Ensembl version 84, Rnor_6.0 GCA_000001895.4) using the STAR Aligner v2.3^56^. Read counts were quantified using the feature counts software package^57^. Differential gene expression was calculated with the Bioconductor LIMMA analysis R package on voom normalized read counts^58^. Significantly differentially expressed genes in CDAA vs. CSAA conditions were filtered using an adjusted *p* value cutoff of 0.01 (after Benjamini–Hochberg multiple testing correction^59^) and a log_2_ fold change of > = |1|. For principal component analysis of FPKM normalized mRNA-Seq data, we only kept genes with at least one read count (n = 20,868), leaving a small proportion of missing values (0.25%). PCA was performed using R software^60^ with the probabilistic PCA (ppca) function implemented in the pcaMethods package^61^ on the log_2_ transformed and mean centered data. Standardized (*z*-score) log ratios of transcript time course data were clustered using Euclidean distance and average linking. Overrepresentation analysis of GO categories for Molecular Function, Cellular Component, and Biological Process in individual clusters was done in the Perseus software^62^ using all expressed genes as background (Fisher exact test with Benjamini Hochberg multiple testing correction). We defined genes as expressed which had ≥1CPM (one count per million mapped reads) in the majority of samples in at least one experimental group.

Tissue specificity and enrichment has been determined using RNA-Seq data from two published data sets: 1) A reference panel of 27 human normal tissues including whole liver as described in^63^ (ArrayExpress E-MTAB-1733) and 2) an assay of hepatic stellate cells which have been activated *in vitro* using TGFb as described in^64^ (Gene Expression Omnibus GSE78853). RNA-Seq raw data has been processed as described above. For each normal tissue and the untreated and treated hepatic stellate cell, the geometric mean FPKM expression was determined. The median expression across all tissues was determined from the geometric mean of median values. The enrichment factor for each tissue and the two hepatic stellate cell conditions was then determined by the fold change of median expression per tissue vs. geometric mean across all tissues.

#### Proteomics data analysis

Data were analyzed with Proteome Discoverer 2.1 (Thermo Fisher Scientific). All data were searched against a composite Ensembl target/decoy database for *Rattus norvegicus* (Ensembl version 84, Rnor_6.0 GCA_000001895.4) using the SEQUEST algorithm^65^. MS2 spectra were searched using 10 ppm precursor tolerance and 0.6 Da tolerance for fragments, allowing 2 missed cleavages. Oxidation of methionine, acetylation of protein N-terminus and phosphorylation of serine, tyrosine and threonine were set as dynamic modifications. The peptide spectrum matches were filtered at a Percolator FDR of 1%^66^.

TMT reporter ion signal-to-noise values were quantified from MS3 scans using a 10 ppm integration tolerance with the most confident centroid setting, a co-isolation threshold of 50 and an average reporter S/N threshold of 1. Quantitative data was normalized on total peptide amount and scaled to channel average of 100.

#### Further proteomics data processing and downstream analysis

First, data from the two individual runs were filtered to exclude contaminants and proteins with low FDR confidence (>0.01). To combine the two runs the data were filtered for having at least six out of eight replicates of one experimental group, leaving n = 3,273 in the liver data and n = 433 in the plasma dataset for further analysis.

Data analysis was done in Perseus software^62^. Data were log_2_ transformed and missing values replaced (liver 1.5%, plasma 0.5%) by sampling from log normal distribution with a downshift of 2.5 and width of 0.2 for the liver dataset and 1.8 downshift and 0.3 width or the plasma dataset relative to the log normal distribution. Parameters were optimized using PCA towards maximizing variance in PC1 (separating NASH vs healthy samples). Finally, to remove batch effects the data of each experimental run were mean centered. Sample 906_CSAA was removed from further analysis as it represented a clear outlier as judged by hierarchical clustering and principal component analysis. To assess statistical significance we performed a two sided T-test using the permutation based FDR, with a cutoff at 0.01. For comparison to RNA data, we mapped all rat proteins to corresponding rat genes using the Ensembl database.

#### Sample level correlation with liver fibrosis

We linked gene and protein expression at the sample level to the observed degree of fibrosis by linear correlation analysis. For gene expression, we used normalized gene expression level per gene per (FPKM) and for protein expression, we used relative intensities per identified protein per sample. For degree of fibrosis, we used the fibrosis scores per animal as described in^19^.

#### Ingenuity pathway analysis

Core analyses of transcriptomics and proteomics datasets were performed with Ingenuity Pathway Analysis (IPA) platform (QIAGEN N.V., Venlo, NL) using the standard settings. Canonical pathways and hepatotoxicity functions were filtered to *z*-scores and *p*-values as indicated in the figures.

#### Ranking approach for biomarker candidate selection

Potential soluble biomarker candidates were ranked using a multi-linear weighted sum equation according to

1 $$scor{e}_{total,j}={\sum }_{j=1}^{m}{w}_{j}\ast sub\_scor{e}_{i,j}$$

whereby 0 < = *wj* < = 1 corresponds to the weight *j* for the *sub_score j* of the gene *i*. We mapped all rat proteins to corresponding rat genes and all rat genes to next human orthologues using the Ensembl database (Version 84). Furthermore, we then mapped all human genes to known human proteins as referenced by the UniProt/Swissprot database from 2018–04–24. For each gene and corresponding protein, we compiled subscores from experimental data and public databases and mapped them to four different ranking dimensions (see Table 1):

1. Plasma specificity (differential protein expression observed in plasma, sample level correlation with liver fibrosis)
2. Liver specificity for both RNA and protein data (differential expression as well as sample level correlation with liver fibrosis)
3. Prior evidence (association to fibrosis as given by OpenTargets overall score (https://opentargets.org downloaded on 2018-07-24), NASH literature acceding to GeneRifs from EntrezGene and co-occurrence in Pubmed abstract, patent US2014/0303018A1 (Tables 2–7 claiming 39 plasma biomarkers for NASH) and genes coding for biomarkers for NASH according to MetaCoreClarivate Analytics database (MetaCore Version from 2017-02-14, see https://clarivate.com)
4. Secreted gene protein product according to the UniProt/Swissprot database.

For each dimension, subscores were derived using again Eq. (1). We calculated differential expression subscores for RNA and protein from *p*-value and log_2_ fold change in CDAA vs. CSAA contrasts according to

2 $$dge{\_}_{score}(contras{t}_{i,k})=-\,\log \,10(padj(contras{t}_{i,k})\ast |\,\log \,2(contras{t}_{i,k})|$$

whereby *k* corresponds to the contrast *k* (i.e. plasma protein, liver protein or liver RNA). Feature subscores for histology correlation have been determined using the square of the Pearson correlation coefficient between RNA expression FPKM and protein intensity values, respectively. Subscores for literature evidence were derived from mean of rank normalized scores for publication counts and publication counts divided by expected counts (based on all publications referencing the gene or protein name). A complete list of dimension subscores and features is shown in Table 1.

Dimension weights were set *a priori*. The sensitivity of total scores was assessed by applying a number of different weight settings referred to as “default”, “prior evidence”, “balanced”, “liver/plasma specific”, “liver specific” and “plasma specific” ranking. Corresponding weight settings are listed in Table 3.

## Supplementary information

Supplementary Table S1.

Supplementary Table S2.

Supplementary Table S3.

## Data Availability

The RNA-Seq dataset generated during the current study are available in the Gene Expression Omnibus, https://www.ncbi.nlm.nih.gov/geo/query/acc.cgi?acc=GSE134715. The mass spectrometry proteomics data have been deposited to the ProteomeXchange Consortium via the PRIDE^67^ partner repository with the dataset identifier PXD014751.
